# Facing Frailty: Exploring the Effectiveness of Integrated Care for Frail Older People: Erasmus School of Health Policy & Management, Erasmus University, 2018, ISBN 978-94-6361-178-7

**DOI:** 10.5334/ijic.4736

**Published:** 2019-08-01

**Authors:** Willemijn M. Looman

**Affiliations:** 1 Erasmus School of Health Policy and Management, Erasmus University Rotterdam, NL

**Keywords:** integrated care, frail older people, primary care, effectiveness, cost-effectiveness, prevention

## Abstract

This thesis aimed to explore the (cost-)effectiveness of preventive, integrated care for community-dwelling frail older people. The first part of this thesis focused on the effectiveness and cost-effectiveness of a specific preventive integrated care intervention, the Walcheren Integrated Care Model (WICM). The second part of this thesis critically reflected on the concepts and methodologies used to explore the (cost-)effectiveness of integrated care for frail older people. This second part included a systematic review and an exploration of the effectiveness of integrated care for six profiles of frail older people.

## Outline

Due to population ageing, primary care systems throughout the world are encountering great challenges urging innovation in the organization of elderly care. As frail older people suffer from problems in the physical, psychological and social domain, primary care professionals struggle with this increasing complexity and perceive difficulties in providing high quality care. Currently, care for frail older people is often reactive, fragmented and lacks coordination and to overcome these barriers, integrated care is considered a promising solution. Professionals, policy makers and researchers have high expectations of integrated care and the wide range of aims it is has been claimed to achieve. To assess whether integrated care can meet these high and diverse expectations, this thesis aimed to explore the (cost-)effectiveness of preventive, integrated care for community-dwelling frail older people.

## Introduction

**Figure 1:**
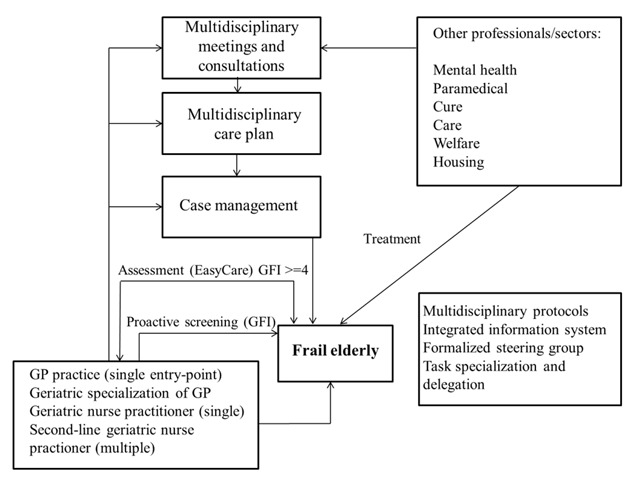
Walcheren Integrated Care Model.

The first part of this thesis focused on the Walcheren Integrated Care Model (WICM) (see Figure 1 and [1]) that was implemented in three GP practices in Walcheren, a region in the south-west of the Netherlands. The WICM is a comprehensive intervention including frailty screening, geriatric assessments, case management, multidisciplinary teams, a single entry point, multidisciplinary protocols and discussions, web-based patient files, and a provider network. The evaluation study of the WICM had a quasi-experimental design with before and after measurements, at three and twelve months (n = 377 frail older people) [2,3,4].

The second part of this thesis critically reflected on the concepts and methodologies used to explore the (cost-)effectiveness of integrated care for frail older people. First, a systematic review was performed including 46 studies on the (cost-)effectiveness of 29 integrated care interventions for frail older people. Different elements and levels of integration – adapted from Valentijn’s Rainbow Model of Integrated Care – were related to the outcomes of integrated care [5]. Second, the heterogeneity within the population of frail older people was further explored by developing frailty profiles with latent class analysis based on the data of 43,000 older people [6]. Subsequently, these frailty profiles were included as subgroups in an individual-patient-data analysis of eight integrated care interventions in Dutch primary care to explore whether integrated care is more effective for specific profiles of frail older people.

## Results and findings

The results of the WICM were in line with the outcomes of other preventive, integrated care interventions included in the systematic review. The results revealed that the majority of the outcomes in the studies on preventive, integrated care showed no significant effects. The effects on health outcomes and functional abilities were limited, whilst well-being appeared to be positively affected. The WICM, for example, had a positive effect on the dimension love and friendship and had a moderately positive effect on general quality of life. Care process outcomes were also promising since they improved for preventive, integrated care interventions. Overall, health care utilization showed mixed results and the evidence for cost-effectiveness was limited. The WICM was also not cost-effective due to the higher costs and the limited effects of the intervention in terms of health-related quality of life. Moreover, the systematic review showed that there is no clear relation between the elements and levels of integrated care and the outcomes of integrated care.

In the second part of this thesis, six frailty profiles were distinguished: *relatively healthy, mild physically frail, psychologically frail, severe physically frail, medically frail* and *multi-frail*. The results thus showed that thé frail older person does not exist and that specific patterns underlie the problems in the different domains of functioning of frail older people. However, for none of these six profiles integrated care was effective on health outcomes. Yet, the results indicated that when the type and severity of the problems of frail older people, and thereby the complexity, increases, the effects of integrated care also vary increasingly.

## Implications for integrated care

This thesis showed that the effects of integrated care for frail older people do not fully meet the high and diverse expectations. Based on our findings, integrated care interventions should be aligned more properly to the target population of frail older people. The heterogeneity of frail older people has increased since the conceptualization of frailty now also includes the psychological and social domain in addition to the physical domain. However, the underlying assumptions concerning effective integrated interventions have not adapted to this changed definition of frailty. Integrated care interventions in primary care settings remain characterized by medical dominance, whereas, research indicates that a more holistic and person-centered approach is required. Moreover, the thesis argues that prevention should be integrated more carefully within the integrated care interventions which could be achieved by a stronger focus on self-management and on the abilities to adapt or to cope with deterioration in health and well-being. Furthermore, the outcome measures used should be better aligned to frail older people and go beyond traditional health outcomes by including well-being and resilience.

Finally, the thesis argues that effective integrated care requires research that is integrated, continuous, and person-centred to cover the complexities of daily practice. Bridges should be built between research, practice and policy and researchers investigating integrated care should work together more closely. Continuity could be improved by exchanging knowledge and context-based evidence between practice and research more quickly in order to keep learning continuously. As this thesis showed that ‘thé frail older person’ does not exist, our focus should be on person-centeredness within integrated care interventions *and* research.

The results presented in this review are based on the author’s thesis presented at Erasmus University Rotterdam on 14 December 2018.
